# Sociodemographic and geographic characteristics associated with patient visits to osteopathic physicians for primary care

**DOI:** 10.1186/1472-6963-11-303

**Published:** 2011-11-04

**Authors:** John C Licciardone, Karan P Singh

**Affiliations:** 1 The Osteopathic Research Center, University of North Texas Health Science Center-Texas College of Osteopathic Medicine, 3500 Camp Bowie Boulevard, Fort Worth, TX 76107, USA; 2 Department of Biostatistics, University of North Texas Health Science Center-School of Public Health, 3500 Camp Bowie Boulevard, Fort Worth, TX 76107, USA

## Abstract

**Background:**

Health care reform promises to dramatically increase the number of Americans covered by health insurance. Osteopathic physicians (DOs) are recognized for primary care, including a "hands-on" style with an emphasis on patient-centered care. Thus, DOs may be well positioned to deliver primary care in this emerging health care environment.

**Methods:**

We used data from the National Ambulatory Medical Care Survey (2002-2006) to study sociodemographic and geographic characteristics associated with patient visits to DOs for primary care. Descriptive analyses were initially performed to derive national population estimates (NPEs) for overall patient visits, primary care patient visits, and patient visits according to specialty status. Osteopathic and allopathic physician (MD) patient visits were compared using cross-tabulations and multiple logistic regression to compute odds ratios (ORs) and 95% confidence intervals (CIs) for DO patient visits. The latter analyses were also conducted separately for each geographic characteristic to assess the potential for effect modification based on these factors.

**Results:**

Overall, 134,369 ambulatory medical care visits were surveyed, representing 4.6 billion (NPE) ± 220 million (SE) patient visits when patient visit weights were applied. Osteopathic physicians provided 336 million ± 30 million (7%) of these patient visits. Osteopathic physicians provided 217 million ± 21 million (10%) patient visits for primary care services; including 180 million ± 17 million (12%) primary care visits for adults (21 years of age or older) and 37 million ± 5 million (5%) primary care visits for minors. Osteopathic physicians were more likely than MDs to provide primary care visits in family and general medicine (OR, 6.03; 95% CI, 4.67-7.78), but were less likely to provide visits in internal medicine (OR, 0.37; 95% CI, 0.24-0.58) or pediatrics (OR, 0.21; 95% CI, 0.11-0.40). Overall, patients in the pediatric and geriatric ages, Blacks, Hispanics, and persons in the South and West were less likely to utilize DOs, although there was some evidence of effect modification according to United States Census region.

**Conclusions:**

Health care reform provides unprecedented opportunities for DOs to reach historically underserved populations and to overcome the "pediatric primary-care paradox."

## Background

Osteopathic physicians (DOs) may be overlooked when considering physician workforce issues in the United States. There were 52,827 non-retired DOs nationally in 2006, including postdoctoral trainees, and 59% of practicing DOs were in the specialties of family or general medicine, internal medicine, or pediatrics [1]. Osteopathic physicians provide more than one-third of family and general medicine visits in the Northeast [2]. The Maine Osteopathic Outcomes Study reported that DOs were more likely than allopathic physicians (MDs) to discuss preventive measures and to address such patient-centered issues as the patient's emotional state, family life, and social activities [3]. Osteopathic physicians have identified a caring physician-patient relationship and a "hands-on" style as distinctive elements of the medical care they provide [4].

Shortages of 35,000-44,000 primary care physicians by 2025 have been predicted because of the demands of an increasing and aging adult population in the United States [5]. As health care reform promises to dramatically increase the number of Americans covered by health insurance, greater access to health care will increase the demand for primary care physicians and may further exacerbate the projected shortage. The Patient Protection and Affordable Care Act of 2010 supports several elements aimed at ameliorating this primary care workforce shortage, including increased funding for National Health Services Corps and Title VII health professions programs, grants and graduate medical education funds for primary care residency programs, redistribution of at least 65% of unfilled slots in non-primary care residency programs to primary care or general surgery residency programs, and accelerated pilot testing and implementation of new models such as the patient-centered medical home [6]. However, the lag time for implementation of these strategies and realization of their intended consequences suggests that newly insured patients, particularly in underserved communities, can anticipate difficulties gaining access to primary care [6].

Community health centers are another critical element within health care reform for increasing access to primary care services. Key values of such community health centers - a whole-person orientation, accessibility, affordability, high quality, and accountability - are congruent with the patient-centered medical home concept and could help define a new primary care paradigm in the United States [7]. Osteopathic medicine has been creative in developing innovative community-based curricula, including some wherein all clinical work is based at community health centers [8]. However, community health centers are also vulnerable to the primary care workforce shortage, and to the related problems of limited access for underserved populations and suboptimal quality of primary care graduate medical education, particularly in ambulatory training sites [9].

The findings of a recent evidence-based random simulation model suggest that expanding insurance coverage, even with improved health care quality, would raise costs and worsen health inequity without a concomitant strategy to strengthen primary care capacity and emphasize health protection [10]. Consequently, several lines of evidence indicate that primary care physicians will be critical in meeting the needs of millions of new patients in the reformed health care system, many of whom will emerge from traditionally underserved populations. Nevertheless, although the primary care physician is highly regarded as "a trusted physician who provides comprehensive, continuous care," contracting scope of practice and salary differentials with other specialty physicians are cited as reasons for avoiding this career path [11]. Strategies for countering the projected primary care physician shortages are clearly needed.

The characteristics of DOs suggest that they may play an important role in delivering primary care as health care reform efforts unfold in the United States. We used five-year data from the National Ambulatory Medical Care Survey (NAMCS) to more closely study the sociodemographic and geographic characteristics associated with patient visits to DOs for primary care services.

## Methods

### The National Ambulatory Medical Care Survey

This study was conducted entirely with five-year NAMCS data, from January 2002 through December 2006, acquired from the National Center for Health Statistics. The advantages of using this database are: (1) it provides a representative sample of ambulatory medical care visits throughout the United States; (2) the data are collected and validated using quality checks at several stages within the survey process; (3) the number of ambulatory visits surveyed generally provides substantial statistical power in precisely estimating population parameters and in making valid comparisons between various subgroups of interest; and (4) it provides recent information on ambulatory medical care in the United States.

The concept of the NAMCS to collect data on medical care provided in physician offices in the United States was developed over 30 years ago [12]. Detailed documentation of the NAMCS instrument, methodology, and data files that served as the basis for this study is available elsewhere [13-17]. Patient visits in NAMCS are selected using a multistage probability sample design to reflect ambulatory medical practice nationwide. The sampling frame for NAMCS includes physicians who meet the criteria of being: (1) office-based; (2) principally engaged in patient care activities; (3) nonfederally employed; and (4) not in the specialties of anesthesiology, pathology, or radiology.

### Patient visits and weights

The basic sampling unit for NAMCS is the office-based physician-patient encounter or "patient visit." Each patient visit is assigned a weight based on four factors: (1) probability of being selected by the three-stage sampling design; (2) adjustment for nonresponse; (3) adjustment for physician specialty group; and (4) weight smoothing to minimize the impact of a few physician outliers whose final visit weights are large relative to those for the remaining physicians. These patient visits provide unbiased national population estimates (NPEs) of ambulatory medical care services and facilitate characterization of such services. In some cases, NPEs derived from NAMCS may be unreliable if they are based on fewer than 30 unweighted patient visits or if the relative standard error (standard error [SE] divided by the NPE) is greater than 0.30 [13-17].

### Data collection and processing

Patient record forms (PRFs) are used by participating physicians or their staff to collect NAMCS data for each selected visit. The NAMCS staff perform completeness checks, editing, and quality control measures to ensure the accuracy of PRFs and associated data files. Item nonresponse rates are 5% or less for most variables. Major exceptions relative to this study include race (17% to 27% annual nonresponse rates) and ethnicity (19% to 28% annual nonresponse rates). The NAMCS staff imputed missing data to help compensate for item nonresponse involving several variables of interest within our study. These included birth year (i.e., age), sex, and race in all years from 2002 through 2006, and ethnicity from 2003 through 2006. Imputation was performed by assigning the value from a randomly selected PRF representing another patient with similar known characteristics. Such imputations were performed according to physician specialty, United States Census region (state was used instead of region to impute ethnicity), and primary diagnosis codes.

### Data management and statistical analyses

**Figure 1:**
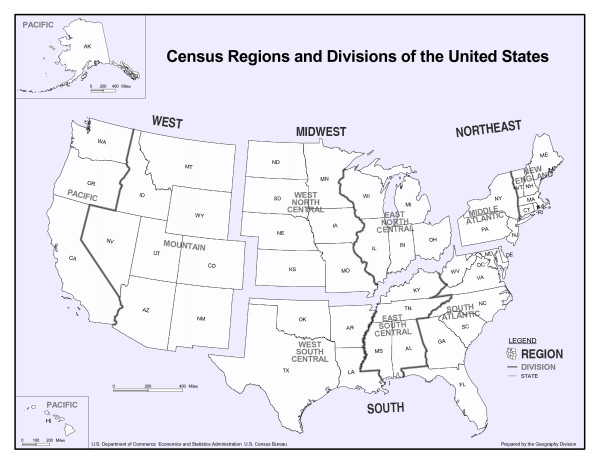
**The aggregation of states and divisions into regions according to the United States Census Bureau**.

Initially, descriptive analyses were performed to derive NPEs for overall patient visits, primary care patient visits, and patient visits according to specialty status. The study focused on measuring utilization of DOs in the provision of ambulatory medical care, particularly with regard to the delivery of primary care services in family and general medicine, internal medicine, and pediatrics. The patient sociodemographic characteristics of interest included age, sex, race, and ethnicity. The geographic characteristics included United States Census region and metropolitan statistical area [MSA] status. As shown in Figure 1, the regions (divisions) in the United States Census are grouped as follows: Northeast (New England, Middle Atlantic); Midwest (East North Central, West North Central); South (South Atlantic, East South Central, West South Central); and West (Mountain, Pacific). Only ambulatory medical care services provided by DOs and MDs were compared because they represent the only two professions that are licensed to practice medicine in the United States and, therefore, these are the only medical practitioners included in the NAMCS. Cross-tabulations and multiple logistic regression were used to compute odds ratios (ORs) and 95% confidence intervals (CIs) for factors associated with patient visits to DOs as compared with MDs, adjusting for potential confounding variables. Patient age, sex, race, ethnicity, United States Census region, and MSA status were forced to enter each logistic regression model. Additionally, multiple logistic regression analyses were conducted separately for each geographic characteristic (i.e., United States Census region and MSA status) to assess the potential for effect modification based on these factors.

The NAMCS data files were merged and analyzed using SPSS for Windows (SPSS Inc., Chicago, IL). Because the multistage probability design of NAMCS includes clustering, stratification, and the assignment of unequal probabilities of selection to sample units, all analyses were performed with the SPSS complex samples module to validly compute NPEs and their SEs [18]. All hypotheses were assessed at the .05 level of statistical significance using two-tailed tests. The Office for the Protection of Human Subjects at the University of North Texas Health Science Center approved this research.

## Results

A total of 134,369 ambulatory medical care visits were surveyed from 2002 through 2006, representing 4.6 billion (NPE) ± 220 million (SE) patient visits when patient visit weights were applied (Table 1). Osteopathic physicians provided ambulatory medical care during 336 million ± 30 million patient visits. These represented 7% of all ambulatory medical care visits in the United States.

**Table 1:** National Ambulatory Medical Care Survey counts and national population estimates for patient visits, according to type of physician*

	Type of physician								
				
	Osteopathic (DO)			Allopathic (MD)			Totals		
			
Type of visit	Survey count	NPE	SE	Survey count	NPE	SE	Survey count	NPE	SE
Overall visits	11,426	336	30	122,943	4,237	204	134,369	4,572	220
Primary care visits	7,190	217	21	39,662	2,021	98	46,852	2,238	109
Family and general medicine†	6,826	212	21	18,750	859	55	25,576	1,071	68
Internal medicine†	947	26	6	8,813	712	47	9,760	737	48
Pediatrics†	351	10	3	12,395	572	33	12,746	582	33

*NPE denotes national population estimate (in millions); SE, standard error (in millions) of the NPE. The NPEs and SEs were computed by applying the appropriate patient visit weights to the actual survey counts. Thus, the 134,369 completed surveys over the five-year period represented an estimated 4.6 billion ± 220 million patient visits throughout the United States in this period.

†The combined NPEs for family and general medicine, internal medicine, and pediatrics exceeded the NPE for primary care because not all physicians in these specialties were primary care physicians.

### Primary care patient visits

Osteopathic physicians provided ambulatory medical care during 217 million ± 21 million patient visits for primary care services, representing 10% of primary care visits from 2002 through 2006. Osteopathic physicians were more likely than MDs to provide primary care visits (OR, 2.07; 95% CI, 1.61-2.67). However, there were important differences in the shares of primary care visits provided by DOs according to specialty, ranging from only 2% for pediatrics, to 3% for internal medicine, and 20% for family or general medicine. Consequently, DOs were more likely than MDs to provide family and general medicine visits (OR, 6.03; 95% CI, 4.67-7.78), but were less likely to provide specialty visits in pediatrics (OR, 0.21; 95% CI, 0.11-0.40) and internal medicine (OR, 0.37; 95% CI, 0.24-0.58). Correspondingly, DOs provided 180 million ± 17 million (12%) of the primary care visits for adults (21 years of age or older) from 2002 through 2006; however, they provided only 37 million ± 5 million (5%) of the primary care visits for minors.

### Sociodemographic and geographic characteristics of patient visits for primary care

Significant differences between DOs and MDs in the age (P < .001), race (P < .001), ethnicity (P = .02), and United States Census region (P < .001) of their primary care patients remained, even after adjusting for potential confounders (Table 2). Patients in the younger (0-14 years and 15-24 years) and older (65-74 years and 75 years or older) age groups, Blacks, and Hispanics were less likely to be seen by DOs. Geographically, patients residing in the South and West regions were less likely to visit DOs than MDs. The overall pattern of utilization of DOs for primary care according to sociodemographic characteristics described above (Table 2) was most evident in the Midwest (Table 3). Additionally, patients in non-MSAs in the Midwest were more likely to visit DOs than MDs. Patients in the youngest age group (0-14 years) were less likely to be seen by DOs during primary care visits across all United States Census regions and in both MSAs and non-MSAs (Table 4). Notably, females in the West were also less likely to utilize DOs than MDs.

**Table 2:** Odds ratios and 95% confidence intervals for sociodemographic and geographic characteristics associated with patient visits to osteopathic physicians for primary care*

	Unadjusted			Adjusted†		
		
Characteristic	OR	95% CI	P	OR	95% CI	P
Age (yrs)			< .001			< .001
< 15	0.33	0.25 - 0.44		0.33	0.25 - 0.45	
15-24	0.77	0.65 - 0.91		0.75	0.64 - 0.88	
25-44	1.00			1.00		
45-64	0.96	0.87 - 1.05		0.94	0.85 - 1.04	
65-74	0.78	0.66 - 0.92		0.76	0.64 - 0.90	
75+	0.70	0.58 - 0.84		0.67	0.56 - 0.81	
						
Sex			.50			.54
Female	1.03	0.95 - 1.11		0.98	0.90 - 1.06	
Male	1.00			1.00		
						
Race			< .001			< .001
White	1.00			1.00		
Black	0.52	0.38 - 0.75		0.55	0.41 - 0.75	
Other	0.38	0.24 - 0.59		0.41	0.24 - 0.69	
						
Ethnicity			< .001			.02
Hispanic	0.55	0.41 - 0.75		0.69	0.51 - 0.94	
Not Hispanic	1.00			1.00		
						
United States Census region			< .001			< .001
Northeast	0.73	0.49 - 1.09		0.77	0.50 - 1.19	
Midwest	1.00			1.00		
South	0.40	0.24 - 0.66		0.41	0.24 - 0.69	
West	0.45	0.30 - 0.68		0.47	0.31 - 0.72	
						
MSA status			.48			.91
MSA	1.00			1.00		
Non-MSA	1.19	0.73 - 1.93		1.03	0.64 - 1.66	

*CI denotes confidence interval; MSA, metropolitan statistical area; OR, odds ratio. ORs and 95% CIs are for osteopathic physicians (DOs) relative to allopathic physicians (MDs).

†The results were computed using logistic regression. The adjusted ORs were based on a multiple logistic regression model that included each of the characteristics in the table.

**Table 3:** Odds ratios and 95% confidence intervals for sociodemographic characteristics and MSA status associated with patient visits to osteopathic physicians for primary care, according to United States Census region*†

	Northeast			Midwest			South			West		
				
Characteristic	OR	95% CI	P	OR	95% CI	P	OR	95% CI	P	OR	95% CI	P
Age (yrs)			< .001			< .001			.01			.03
< 15	0.13	0.08 - 0.20		0.37	0.22 - 0.62		0.44	0.25 - 0.76		0.52	0.30 - 0.91	
15-24	0.57	0.44 - 0.73		0.69	0.52 - 0.93		0.93	0.72 - 1.20		0.93	0.65 - 1.31	
25-44	1.00			1.00			1.00			1.00		
45-64	0.86	0.68 - 1.08		0.88	0.76 - 1.03		0.95	0.81 - 1.12		1.22	0.95 - 1.56	
65-74	0.76	0.49 - 1.17		0.62	0.50 - 0.75		0.88	0.70 - 1.11		1.14	0.79 - 1.63	
75+	0.53	0.34 - 0.85		0.54	0.41 - 0.73		0.81	0.61 - 1.08		1.08	0.65 - 1.81	
												
Sex			.43			.35			.81			.01
Female	0.92	0.75 - 1.13		1.06	0.93 - 1.21		0.99	0.88 - 1.10		0.79	0.66 - 0.94	
Male	1.00			1.00			1.00			1.00		
												
Race			.31			< .001			.04			< .001
White	1.00			1.00			1.00			1.00		
Black	0.64	0.32 - 1.27		0.54	0.32 - 0.93		0.54	0.33 - 0.86		0.66	0.44 - 1.01	
Other	0.67	0.31 - 1.46		0.31	0.18 - 0.52		1.09	0.40 - 2.99		0.26	0.14 - 0.47	
												
Ethnicity			.02			< .001			.88			.18
Hispanic	0.36	0.18 - 0.76		0.57	0.38 - 0.87		1.01	0.61 - 1.67		0.64	0.32 - 1.30	
Not Hispanic	1.00			1.00			1.00			1.00		
												
MSA status			.41			.04			.66			.49
MSA	1.00			1.00			1.00			1.00		
Non-MSA	0.54	0.12 - 2.36		1.74	1.04 - 2.92		0.83	0.36 - 1.91		0.72	0.29 - 1.82	

*CI denotes confidence interval; MSA, metropolitan statistical area; OR, odds ratio. ORs and 95% CIs are for osteopathic physicians (DOs) relative to allopathic physicians (MDs).

†The results were computed using a multiple logistic regression model that included each of the characteristics in the table.

**Table 4:** Odds ratios and 95% confidence intervals for sociodemographic characteristics and United States Census region associated with patient visits to osteopathic physicians for primary care, according to MSA status*†

	MSA			Non-MSA		
		
Characteristic	OR	95% CI	P	OR	95% CI	P
Age (yrs)			< .001			.001
< 15	0.30	0.22 - 0.40		0.53	0.30 - 0.93	
15-24	0.74	0.63 - 0.87		0.81	0.58 - 1.12	
25-44	1.00			1.00		
45-64	0.94	0.85 - 1.04		0.91	0.74 - 1.12	
65-74	0.80	0.66 - 0.96		0.68	0.52 - 0.89	
75+	0.67	0.54 - 0.83		0.62	0.48 - 0.81	
						
Sex			.41			.58
Female	0.96	0.87 - 1.06		0.96	0.83 - 1.11	
Male	1.00			1.00		
						
Race			< .001			.002
White	1.00			1.00		
Black	0.58	0.42 - 0.80		0.41	0.25 - 0.67	
Other	0.40	0.23 - 0.67		1.07	0.53 - 2.18	
						
Ethnicity			.05			.01
Hispanic	0.67	0.49 - 0.93		0.51	0.33 - 0.78	
Not Hispanic	1.00			1.00		
						
United States Census region			.01			.01
Northeast	0.95	0.64 - 1.43		0.34	0.08 - 1.48	
Midwest	1.00			1.00		
South	0.51	0.29 - 0.91		0.26	0.11 - 0.60	
West	0.60	0.40 - 0.89		0.28	0.09 - 0.94	

*CI denotes confidence interval; MSA, metropolitan statistical area; OR, odds ratio. ORs and 95% CIs are for osteopathic physicians (DOs) relative to allopathic physicians (MDs).

†The results were computed using a multiple logistic regression model that included each of the characteristics in the table.

## Discussion

National surveys indicate that less than half of the general public is aware of DOs [19,20]. Additionally, there has been considerable debate about the professional identity and distinctiveness of DOs in the United States [21-25]. Such lack of awareness and ambiguity potentially leads to misperceptions of osteopathic medicine and uncertainty about the role of DOs in the American health care system. This study demonstrated that more than one of every nine adult primary care visits and one of every five primary care visits in the specialty of family or general medicine are provided by DOs. While DOs provide disproportionately more primary care visits than MDs in the United States, the difference between the two professions is entirely attributable to the number of patient visits to DOs in the specialty of family or general medicine, not to DOs in internal medicine or pediatrics. In fact, DOs are less likely than MDs to provide patients visits in the specialty areas of internal medicine or pediatrics. Thus, osteopathic medicine's current professional role may best be described as filling a family or general medicine (i.e., "generalist") niche within health care. Using Porter's generic competitive strategies matrix [26], this generalist role is consistent with the previously described "focused differentiation" strategy for positioning osteopathic medicine within the primary care market sector [27]. This strategy emphasizes promoting the patient-centered practice style of DOs that differentiates them from other health care providers within the focus area of primary care.

This study confirms important differences in the sociodemographic and geographic characteristics of patients seen by DOs and MDs during the provision of primary care services. Osteopathic physicians are generally underutilized by patients at both ends of the age spectrum, Blacks, and Hispanics, although there are some differences in these utilization factors across United State Census regions. The most important, and unexpected, regional difference involves the decreased utilization of DOs among females in the West. The reasons for this finding are unclear and require further elucidation.

Our findings in the pediatric and geriatric age groups are consistent with a relative shortage of DOs within the primary care specialties of pediatrics and internal medicine, respectively [1,28]. Further, a national survey reports that young adults (18-39 years) are significantly less likely than older adults to be aware of DOs [20]. Thus, the decreased ambulatory medical care visits provided by DOs for minors in our study likely reflects both a relative shortage of osteopathic pediatricians and decreased awareness of DOs among the parents of minors (i.e., young adults).

There may be a potentially important generational relationship between awareness and utilization of DOs. Thus, increasing awareness of DOs among young adults may increase the number of pediatric visits for their children provided by DOs. These children, in turn, will be more aware of DOs as they enter adulthood, thereby further enhancing their own utilization of DOs as adults and their children's utilization of DOs for pediatric patient visits. However, in the absence of both a sufficient supply of osteopathic pediatricians to provide primary care services and adequate public awareness of DOs among young adults to drive demand, this "ramp-up" phenomenon will not occur. In the context of a profession perhaps best known for its contributions to primary care [8], we refer to the challenge of simultaneously increasing the supply of osteopathic pediatricians and improving public awareness as osteopathic medicine's "pediatric primary-care paradox."

**Figure 2:**
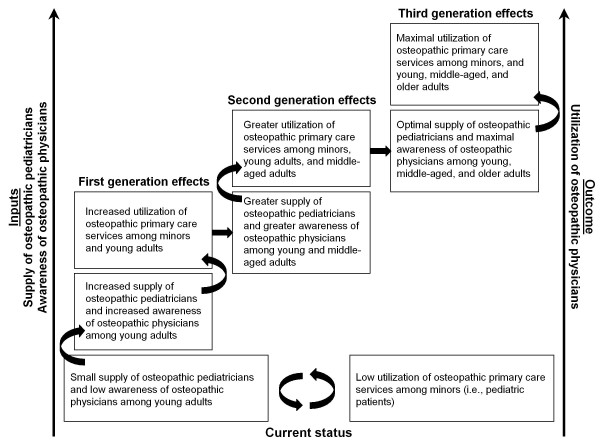
**Proposed resolution of osteopathic medicine's pediatric primary-care paradox**.

An important point relating to "osteopathic identity" involves deciding whether osteopathic medicine retains its generalist focus on family or general medicine, or if it wishes to expand its primary care focus to more broadly include internal medicine, pediatrics, or both. In our opinion, resolving the pediatric primary-care paradox, as illustrated in Figure 2, will be critical to the promotion and long-term growth of osteopathic medicine. However, this will involve sustained efforts and will be a demanding task because of an impending crisis with the current growth in DO and MD college enrollments and the anticipated competition for training in Accreditation Council for Graduate Medical Education (ACGME) postdoctoral programs [29], including those in pediatrics. Assuming no short-term change in the number of graduate medical education positions (including training programs approved by either the American Osteopathic Association or the ACGME), the estimated 24,269 first-year training slots available would be inadequate for the estimated 5,227 osteopathic and 19,909 allopathic graduates in 2012 [30]. This scenario will likely be further exacerbated by another 6,000 or more international medical graduates who may be seeking graduate medical education in the United States [30].

In addition to young adults, members of racial and ethnic minority groups and persons with no more than a high school education are less likely to be aware of DOs, even after adjusting for United States Census region and urbanization of residence [20]. Even among persons claiming to be aware of DOs, members of racial and ethnic minority groups continue to underutilize DOs [20]. These racial and ethnic findings may be partially explained by the underrepresentation of Blacks and Hispanics among the ranks of students at colleges of osteopathic medicine (8% of osteopathic matriculants [31] vs. 14% of allopathic matriculants [32] in 2004). Except for this explanation, however, the reasons for underutilization of DOs by racial and ethnic minority groups are largely unknown. More research is needed to identify and remove barriers to utilization of DOs. Greater efforts are also needed to improve public awareness of DOs among young persons and to attract racial and ethnic minorities to osteopathic medicine, both as patients and physicians.

Osteopathic physician visits are heavily concentrated in the Midwest, with significant underutilization in the South and West. Many, although not all, of our findings may be explained historically by the development of osteopathic medicine in the rural, midwestern United States, thereby creating greater demand for DO services in these areas. Interestingly, while we observed significantly greater utilization of DOs (vs. MDs) by patients in non-MSAs in the Midwest, we found lower (albeit not statistically significant) utilization of DOs in non-MSAs in each of the other United States Census regions. The latter findings are somewhat surprising, as delivery of rural health care is often considered a quintessential feature of osteopathic medical practice.

Growth in the number of colleges of osteopathic medicine, largely located in the Midwest and Northeast during the 20^th ^century, provided an ample supply of DOs in these regions to meet patient demand. Although more recently osteopathic medicine has been innovative in establishing new schools in nontraditional locations [8], a concerted long-term plan for establishing new, geographically-balanced colleges of osteopathic medicine within the range of exemplary, co-located graduate medical education programs is necessary to fill voids in the South and West if osteopathic medicine is to become a vibrant national player in the health care arena. Correspondingly, there is a need to increase the number of primary care visits that DOs provide for female patients in the West.

There are at least three limitations of this study. First, there were substantial missing data for race and ethnicity. While such missing data were imputed by the NAMCS staff, the validity of this approach cannot be directly confirmed. We conducted two parallel analyses that used the five-year NAMCS data to address the issues of missing data and imputation: (1) using only non-imputed race data for 2002 through 2006; and (2) excluding any unknown ethnicity data (ethnicity data may have been recorded by NAMCS as "unknown" in 2002, prior to their imputation of missing ethnicity data from 2003 through 2006). The findings of these parallel analyses did not differ substantively from those reported herein. Second, findings based on fewer than 30 unweighted patient visits or with relative SEs greater than 0.30 may not be statistically reliable [13-17]. Consequently, we limited our analyses to only those variables that generated sufficient survey counts to ensure findings within the acceptable reliability thresholds. Third, the basic sampling unit in NAMCS is the patient visit, not patient. Thus, the extrapolation our findings to patients (rather than patient visits) potentially may be biased if a substantial percentage of repeat patients were included in the NAMCS data and if there were discrepant findings in the repeat patients compared with the non-repeaters. Such bias is extremely unlikely because physician offices were surveyed by NAMCS for only one week and, except for the very smallest practices, systematic random sampling was used to select patient visits during that week.

## Conclusions

Health care reform, with the potential of universal or near-universal coverage in the coming years, may prove to be an unexpected boon for osteopathic medicine that opens access to millions of patients heretofore unfamiliar with osteopathic medicine. To capitalize on these unparalleled opportunities, stakeholders within the osteopathic profession (e.g., national and state osteopathic associations, colleges of osteopathic medicine, osteopathic postdoctoral training institutions, osteopathic physicians, and others) must work collaboratively with their counterparts in the allopathic profession, federal government, and public health agencies to increase public awareness of osteopathic medicine and to promote its primary care role by more fully embracing a focused differentiation strategy to position osteopathic medicine within the primary care market sector. Additionally, these efforts should expand access to the osteopathic primary care workforce among underserved racial and ethnic groups, thereby enhancing the social mission of providing physicians to care for the national population [8]. The emerging health care environment affords osteopathic medicine an opportunity to begin ameliorating its pediatric primary-care paradox. However, osteopathic stakeholders must act nimbly and strategically to seize these opportunities while simultaneously combating the looming crisis in graduate medical education, which soon threatens the availability of adequate postdoctoral training programs for osteopathic medical graduates. In so doing, DOs will move forward and be optimally integrated into the new system of health care in the United States.

## Competing interests

The authors declare that they have no competing interests.

## Authors' contributions

JCL had full access to all of the data in the study and takes responsibility for the integrity of the data and the accuracy of the data analysis. JCL conceived and designed the study. JCL acquired the data. JCL and KPS analyzed and interpreted the data. JCL and KPS drafted the manuscript. JCL and KPS critically revised the manuscript for important intellectual content. JCL obtained funding. JCL and KPS provided administrative, technical, or material support. JCL supervised the study. JCL and KPS have given final approval for publication.

## Acknowledgements

This study was supported by grants to JCL from the Osteopathic Heritage Foundation and the National Institutes of Health-National Center for Complementary and Alternative Medicine (K24AT002422).

The funding bodies provided financial support for the study only and had no role in the design and conduct of the study; the collection, management, analysis, and interpretation of the data; or in the preparation, review, or approval of the manuscript for publication.

## Pre-publication history

The pre-publication history for this paper can be accessed here:

http://www.biomedcentral.com/1472-6963/11/303/prepub

## References

[B1] American Osteopathic Association Fact Sheet 2006 2006 Chicago, IL

[B2] Licciardone JC A comparison of patient visits to osteopathic and allopathic general and family medicine physicians: results from the National Ambulatory Medical Care Survey, 2003-2004 Osteopath Med Prim Care 2007 1 2 10.1186/1750-4732-1-2 17371578 PMC1805772

[B3] Carey TS Motyka TM Garrett JM Keller RB Do osteopathic physicians differ in patient interaction from allopathic physicians? An empirically derived approach J Am Osteopath Assoc 2003 103 313 318 12884943

[B4] Johnson SM Kurtz ME Perceptions of philosophic and practice differences between US osteopathic physicians and their allopathic counterparts Soc Sci Med 2002 55 2141 2148 10.1016/S0277-9536(01)00357-4 12409127

[B5] Colwill JM Cultice JM Kruse RL Will generalist physician supply meet demands of an increasing and aging population? Health Aff (Millwood) 2008 27 w232 241 10.1377/hlthaff.27.3.w232 18445642

[B6] Doherty RB The certitudes and uncertainties of health care reform Ann Intern Med 2010 152 679 682 20378676 10.7326/0003-4819-153-1-201007060-00235

[B7] Adashi EY Geiger HJ Fine MD Health care reform and primary care -- the growing importance of the community health center N Engl J Med 2010 362 2047 2050 10.1056/NEJMp1003729 20427777

[B8] Mullan F Chen C Petterson S Kolsky G Spagnola M The social mission of medical education: ranking the schools Ann Intern Med 2010 152 804 811 20547907 10.7326/0003-4819-152-12-201006150-00009

[B9] Rieselbach RE Crouse BJ Frohna JG Teaching primary care in community health centers: addressing the workforce crisis for the underserved Ann Intern Med 2010 152 118 122 20008743 10.7326/0003-4819-152-2-201001190-00186

[B10] Milstein B Homer J Hirsch G Analyzing national health reform strategies with a dynamic simulation model Am J Public Health 2010 100 811 819 10.2105/AJPH.2009.174490 20299653 PMC2853627

[B11] Brook RH Young RT The primary care physician and health care reform JAMA 2010 303 1535 1536 10.1001/jama.2010.472 20407064

[B12] Tenney JB White KL Williamson JW National Ambulatory Medical Care Survey: Background and Methodology 1974 Hyattsville, MD: National Center for Health Statistics Vol Series 2, No. 61 25102417

[B13] National Center for Health Statistics 2002 NAMCS Micro-Data File Documentation ftp://ftp.cdc.gov/pub/Health_Statistics/NCHS/Dataset_Documentation/NAMCS/doc02.pdf

[B14] National Center for Health Statistics 2003 NAMCS Micro-Data File Documentation ftp://ftp.cdc.gov/pub/Health_Statistics/NCHS/Dataset_Documentation/NAMCS/doc03.pdf

[B15] National Center for Health Statistics 2004 NAMCS Micro-Data File Documentation ftp://ftp.cdc.gov/pub/Health_Statistics/NCHS/Dataset_Documentation/NAMCS/doc04.pdf

[B16] National Center for Health Statistics 2005 NAMCS Micro-Data File Documentation ftp://ftp.cdc.gov/pub/Health_Statistics/NCHS/Dataset_Documentation/NAMCS/doc05.pdf

[B17] National Center for Health Statistics 2006 NAMCS Micro-Data File Documentation ftp://ftp.cdc.gov/pub/Health_Statistics/NCHS/Dataset_Documentation/NAMCS/doc06.pdf

[B18] Siller AB Tompkins L The big four: analyzing complex sample survey data using SAS^®^, SPSS^®^, STATA^®^, and SUDAAN^® ^(Paper 172-31). 2006 Cary, NC Paper presented at: Proceedings of the Thirty-first Annual SAS^® ^Users Group International Conference.

[B19] Licciardone JC Herron KM Characteristics, satisfaction, and perceptions of patients receiving ambulatory healthcare from osteopathic physicians: a comparative national survey J Am Osteopath Assoc 2001 101 374 385 11476027

[B20] Licciardone JC Awareness and use of osteopathic physicians in the United States: results of the Second Osteopathic Survey of Health Care in America (OSTEOSURV-II) J Am Osteopath Assoc 2003 103 281 289 12834101

[B21] Gevitz N Sectarian medicine JAMA 1987 257 1636 1640 10.1001/jama.257.12.1636 3546754

[B22] Eckberg DL The dilemma of osteopathic physicians and the rationalization of medical practice Soc Sci Med 1987 25 1111 1120 10.1016/0277-9536(87)90352-2 3686076

[B23] Meyer CT Price A The crisis in osteopathic medicine Acad Med 1992 67 810 816 10.1097/00001888-199212000-00002 1457012

[B24] Gevitz N 'Parallel and distinctive': the philosophic pathway for reform in osteopathic medical education J Am Osteopath Assoc 1994 94 328 332 8027001

[B25] Gevitz N Center or periphery? The future of osteopathic principles and practices J Am Osteopath Assoc 2006 106 121 129 16585378

[B26] Porter ME Competitive Strategy: Techniques for Analyzing Industries and Competitors 1980 New York: The Free Press

[B27] Licciardone JC Osteopathic research: elephants, enigmas, and evidence Osteopath Med Prim Care 2007 1 7 10.1186/1750-4732-1-7 17371583 PMC1808471

[B28] Bureau of Labor Statistics - U.S. Department of Labor Physicians and surgeons Occupational Outlook Handbook, 2010-11 Edition

[B29] Cummings M Sefcik DJ The impact of osteopathic physicians' participation in ACGME-accredited postdoctoral programs, 1985-2006. Acad Med 2009 84 733 736 10.1097/ACM.0b013e3181a3de21 19474548

[B30] Kane GC Grever MR Kennedy JI The anticipated physician shortage: meeting the nation's need for physician services Am J Med 2009 122 1156 1162 10.1016/j.amjmed.2009.07.010 19958898

[B31] American Association of Colleges of Osteopathic Medicine 2006 Annual Statistical Report on Osteopathic Medical Education 2007 Chevy Chase, MD

[B32] Association of American Medical Colleges More Apply to U.S. Medical Schools: Minority Enrollment Increases After Downturn in 2003 2004 Washington, DC

